# Supplementary material for: Unraveling the In Vivo Protein Corona
Source: Cells. 2021 Jan 12;10(1):132. doi: 10.3390/cells10010132 (PMC7826990; doi:10.3390/cells10010132)
Supplement: Supplementary file 1 [file cells-10-00132-s001.pdf]

Supplementary Information

1. Depletion of Macrophages In Vivo

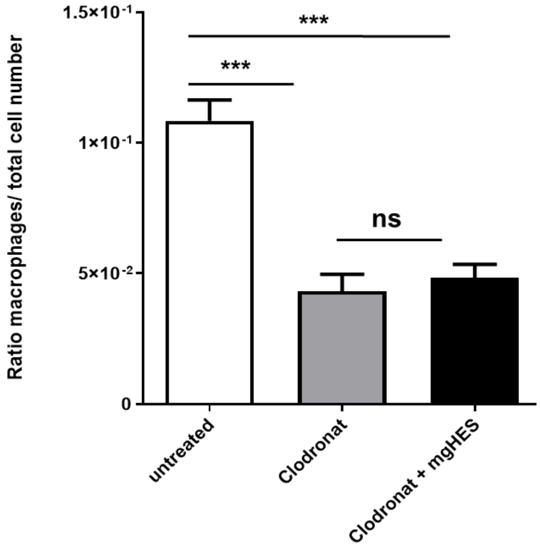

Figure S1. Depletion of macrophages in vivo using clodronate-lipsomes. The ratio between F480$^+$ cells (macrophage) and total cell number was calculated. For statistical analysis a two-way ANOVA test was performed. *ns* = not significant, $p < 0.05$* $p < 0.01$** $p < 0.001$*** ($n = 12$ for un-treated, $n = 8$ for clodronate treated $n = 34$ for clodronate + mgHES treated).

2. In Vitro Protein Corona Analysis

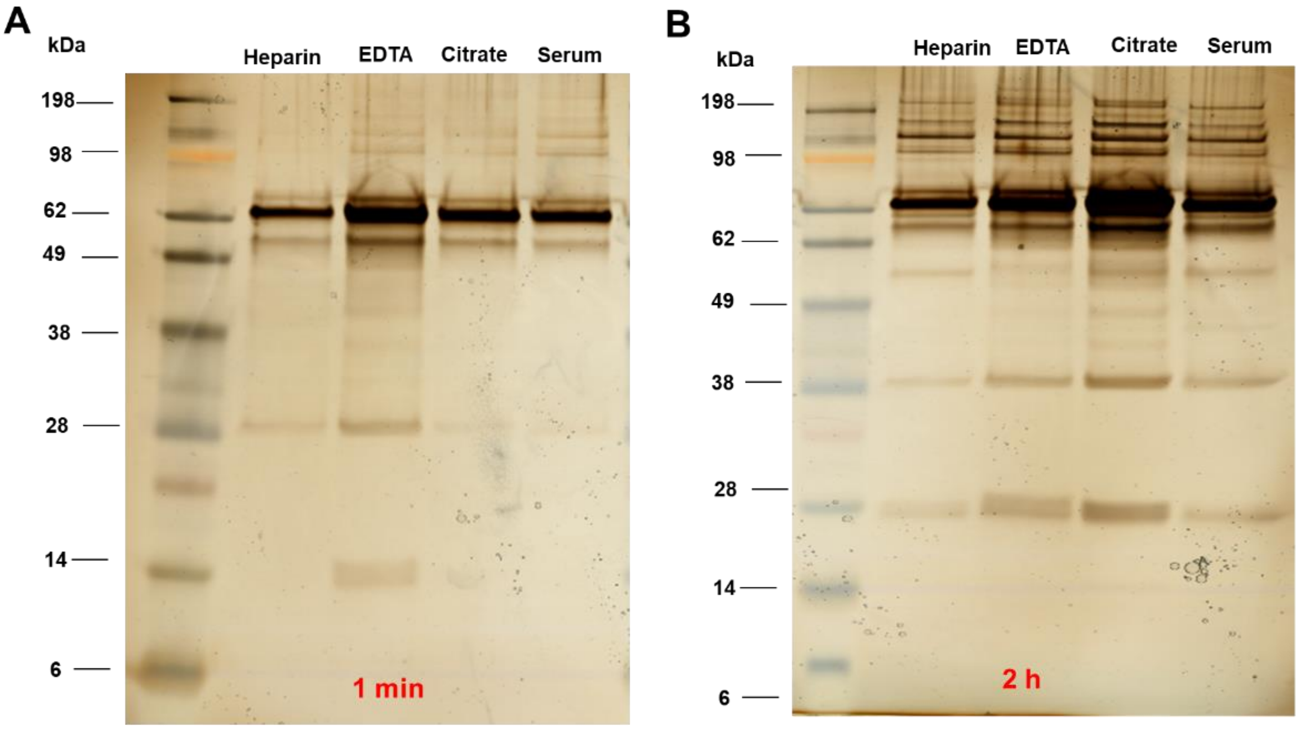

Figure S2. mgHES nanoparticles were incubated with plasma or serum for 1 min (**A**) or 2 h (**B**). The protein corona was purified via magnetic separation. Proteins were desorbed from the nanoparticle surface and visualized by SDS PAGE.

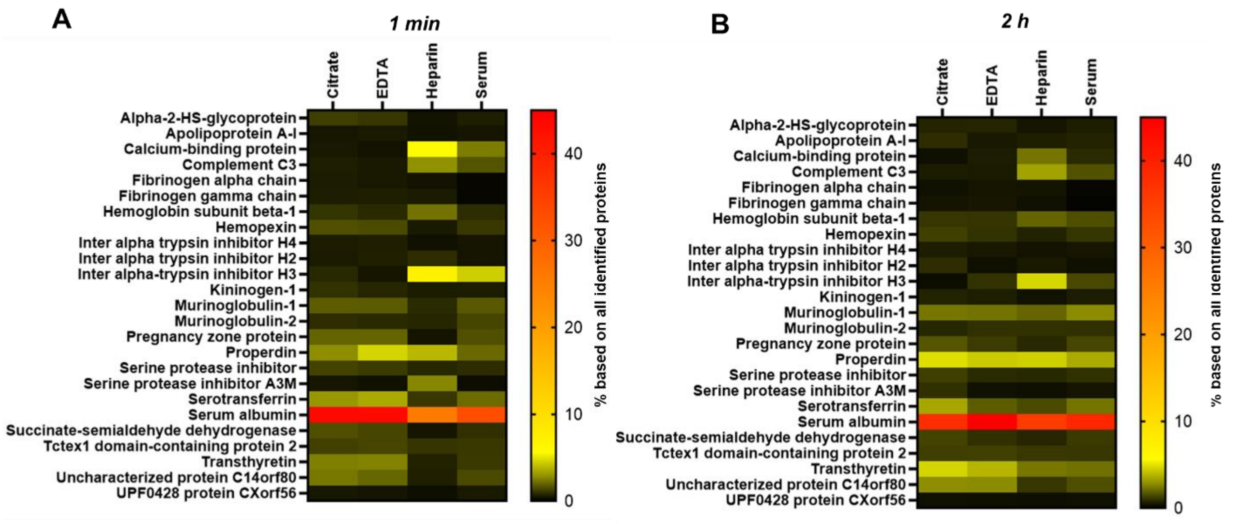

Figure S3. Protein corona proteins after 1 min (**A**) or 2 h (**B**) in vitro incubation were analysed by LC-MS. The most abundant proteins (25) are summarized in the heat map.

Table S1. *p* values for all corona proteins shown in Figure S3. *p* values were determined for each protein source individually and the amount of each corona protein was compared for the two time points (1 min vs. 2 h).

Citrate		p value	EDTA		p value
Alpha-2-HS-glycoprotein	*	0,0222	Alpha-2-HS-glycoprotein	ns	0,9983
Apolipoprotein A-I	ns	0,518	Apolipoprotein A-I	ns	>0,9999
Calcium-binding protein	ns	0,2388	Calcium-binding protein	ns	>0,9999
Complement C3	ns	0,9967	Complement C3	ns	>0,9999
Fibrinogen alpha chain	ns	0,199	Fibrinogen alpha chain	ns	>0,9999
Fibrinogen gamma chain	ns	0,1127	Fibrinogen gamma chain	ns	>0,9999
Hemoglobin subunit beta-1	ns	>0,9999	Hemoglobin subunit beta-1	ns	>0,9999
Hemopexin	ns	0,2691	Hemopexin	ns	0,8628
Inter alpha trypsin inhibitor H4	ns	0,3193	Inter alpha trypsin inhibitor H4	ns	>0,9999
Inter alpha trypsin inhibitor H2	ns	0,4597	Inter alpha trypsin inhibitor H2	ns	0,9993
Inter alpha-trypsin inhibitor H3	ns	0,0567	Inter alpha-trypsin inhibitor H3	ns	0,6826
Kininogen-1	*	0,0376	Kininogen-1	ns	>0,9999
Murinoglobulin-1	ns	0,7448	Murinoglobulin-1	ns	0,9169
Murinoglobulin-2	ns	0,9708	Murinoglobulin-2	ns	>0,9999
Pregnancy zone protein	ns	0,2949	Pregnancy zone protein	ns	0,1566
Properdin	ns	0,5112	Properdin	ns	>0,9999
Serine protease inhibitor A3K	ns	0,998	Serine protease inhibitor A3K	ns	0,9989
Serine protease inhibitor A3M	*	0,0213	Serine protease inhibitor A3M	ns	>0,9999
Serotransferrin	ns	0,1671	Serotransferrin	****	<0,0001
Serum albumin	**	0,0063	Serum albumin	***	0,0002
Succinate dehydrogenase	ns	0,9814	Succinate dehydrogenase	ns	0,972
Tctex1 domain-containing protein 2	ns	>0,9999	Tctex1 domain-containing protein 2	ns	>0,9999
Transthyretin	**	0,0019	Transthyretin	*	0,0215
Uncharacterized protein C14orf80	ns	0,2675	Uncharacterized protein C14orf80	ns	0,293
UPF0428 protein CXorf56	ns	0,1032	UPF0428 protein CXorf56	ns	>0,9999

Heparin		p value	Serum		p value
Alpha-2-HS-glycoprotein	ns	>0,9999	Alpha-2-HS-glycoprotein	ns	>0,9999
Apolipoprotein A-I	ns	>0,9999	Apolipoprotein A-I	****	<0,0001
Calcium-binding protein	ns	0,9482	Calcium-binding protein	****	<0,0001
Complement C3	ns	>0,9999	Complement C3	ns	0,9999
Fibrinogen alpha chain	ns	>0,9999	Fibrinogen alpha chain	ns	>0,9999
Fibrinogen gamma chain	ns	>0,9999	Fibrinogen gamma chain	ns	>0,9999
Hemoglobin subunit beta-1	ns	>0,9999	Hemoglobin subunit beta-1	****	<0,0001
Hemopexin	ns	>0,9999	Hemopexin	ns	0,9979
Inter alpha trypsin inhibitor H4	ns	>0,9999	Inter alpha trypsin inhibitor H4	ns	>0,9999
Inter alpha trypsin inhibitor H2	ns	>0,9999	Inter alpha trypsin inhibitor H2	ns	0,7761
Inter alpha-trypsin inhibitor H3	ns	0,9991	Inter alpha-trypsin inhibitor H3	****	<0,0001
Kininogen-1	ns	>0,9999	Kininogen-1	ns	>0,9999
Murinoglobulin-1	ns	>0,9999	Murinoglobulin-1	****	<0,0001
Murinoglobulin-2	ns	>0,9999	Murinoglobulin-2	****	<0,0001
Pregnancy zone protein	ns	>0,9999	Pregnancy zone protein	*	0,0149
Properdin	ns	>0,9999	Properdin	****	<0,0001
Serine protease inhibitor A3K	ns	>0,9999	Serine protease inhibitor A3K	ns	0,904
Serine protease inhibitor A3M	*	0,0337	Serine protease inhibitor A3M	ns	0,0888
Serotransferrin	ns	>0,9999	Serotransferrin	*	0,0413
Serum albumin	****	<0,0001	Serum albumin	****	<0,0001
Succinate dehydrogenase	ns	>0,9999	Succinate dehydrogenase	**	0,0079
Tctex1 domain-containing protein 2	ns	>0,9999	Tctex1 domain-containing protein 2	ns	>0,9999
Transthyretin	ns	0,9975	Transthyretin	****	<0,0001
Uncharacterized protein C14orf80	ns	>0,9999	Uncharacterized protein C14orf80	ns	0,5481
UPF0428 protein CXorf56	ns	>0,9999	UPF0428 protein CXorf56	*	0,0413

3. In VIVO Protein corona analysis

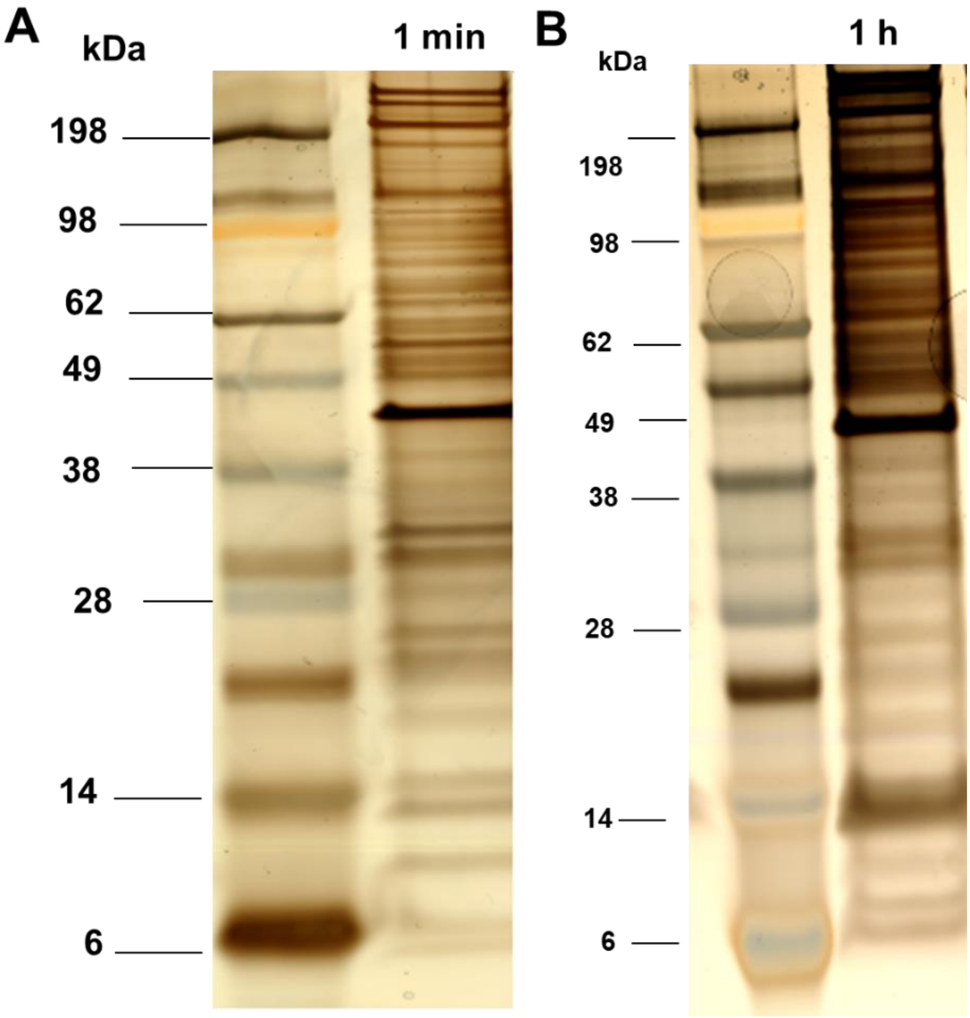

Figure S4. mgHES nanoparticles were injected into mice, the blood was isolated after distinct time points (**A** = 1 min, **B** = 1 h) and nanoparticles were recovered via magnetic separation. The protein corona pattern was visualized by SDS-PAGE.

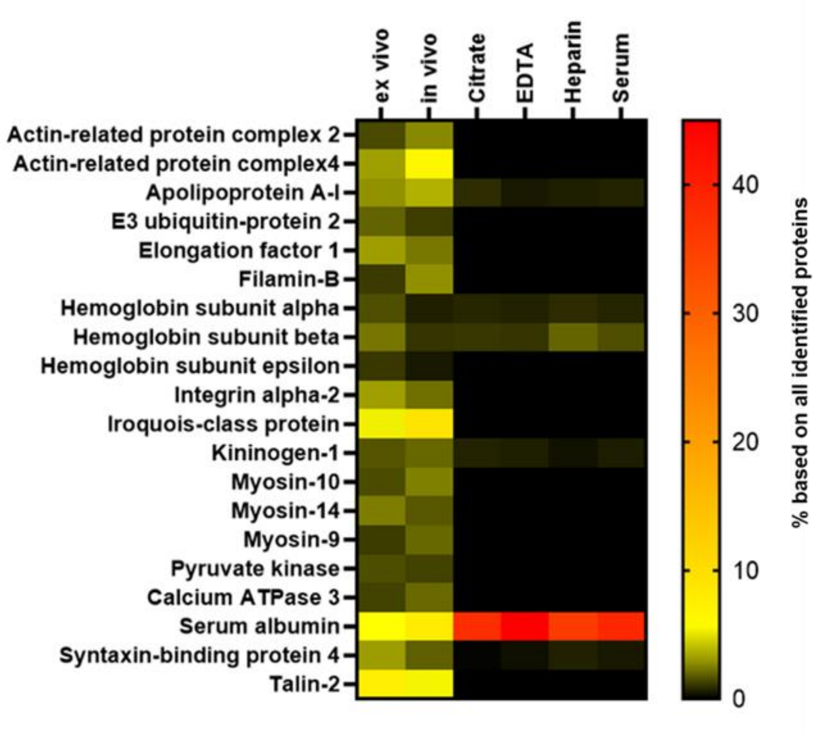

Figure S5. To mimic in vivo corona formation, nanoparticles were incubated directly in blood (ex vivo) for 1 min. The heat map of the 20 most abundant corona proteins highlights the similarity between the ex vivo and in vivo corona after 1 min of blood circulation. The average amount of each protein in % is shown and calculated from technical triplicates and biological replicates for ex vivo (n = 2) and in vivo (n = 5). For comparison nanoparticles were incubated with serum or plasma for 2 h. All identified proteins are summarized in a separate Excel Sheet.
